# Mitomycin intravascular chemoembolization (MICE) to treat corneal vascularization prior to penetrating keratoplasty

**DOI:** 10.1016/j.ajoc.2024.101993

**Published:** 2024-01-14

**Authors:** Neal Rangu, Kamran M. Riaz

**Affiliations:** a Department of Ophthalmology, Dean McGee Eye Institute, University of Oklahoma Health Sciences Center, Oklahoma City, OK, USA; b College of Medicine, University of Oklahoma, Oklahoma City, OK, USA

**Keywords:** Mitomycin intravascular chemoembolization, Corneal neovascularization, Penetrating keratoplasty

## Abstract

**Purpose:**

To present the clinical outcome of mitomycin intravascular chemoembolization (MICE) as a prophylactic treatment in a patient with HSV-induced corneal neovascularization (NV) before penetrating keratoplasty (PKP).

**Observations:**

A 53-year-old male patient presented with a medical history of recurrent herpes simplex virus (HSV) corneal infection. The patient reported worsening visual acuity despite maintenance treatment with systemic antivirals and topical corticosteroids. After the appearance of corneal NV, subconjunctival triamcinolone and bevacizumab injections were given with limited and temporary improvement. Due to worsening corneal NV, MICE was subsequently performed, resulting in the elimination of corneal NV from the visual axis, which allowed for successful PKP 4 months later. Cataract surgery was performed 6 months after PKP.

**Conclusions and importance:**

This report describes the potential efficacy of MICE as a prophylactic treatment for corneal NV prior to PKP.

## 1 Introduction

Corneal neovascularization (NV) is a significant contributor to corneal blindness and can arise from various underlying causes, such as infection, rejection, limbal stem cell deficiency, trauma, ischemia, and mechanical injury.^1^^,^^2^ The associated mechanisms of corneal NV-induced corneal blindness include irregular astigmatism, lipid deposition, stromal scarring, and loss of corneal sensation.^1^ While treatment of corneal NV is focused on addressing the primary cause of vascular proliferation, results of traditionally utilized options have been limited at best.^3^ Topical corticosteroids have shown limited evidence in reversing corneal vascularization.^4^ Other treatment options, including fine needle diathermy (FND), photodynamic therapy, argon laser, and anti-vascular endothelial growth factor (VEGF) treatments, have resulted in a similar lack of long-term success.^1^, ^2^, ^3^^,^^5^ Corneal transplantation options, such as penetrating keratoplasty (PKP), have a high rate of graft failure and rejection risk in corneal NV eyes.^6^ Recently, Ouano introduced a novel approach of using mitomycin C (MMC) intravascular chemoembolization (MICE) as a primary treatment for corneal NV-associated lipid keratopathy, with promising results in three patients.^7^ While MICE is effective for treating corneal NV, pre-existing and subsequent corneal scarring may remain, causing decreased visual acuity and requiring additional surgical interventions. We surmised that MICE can be used as a prophylactic therapy to treat corneal NV in high-risk eyes to increase the chances of surgical success with PKP.

## 2 Case report

**Fig. 1:**
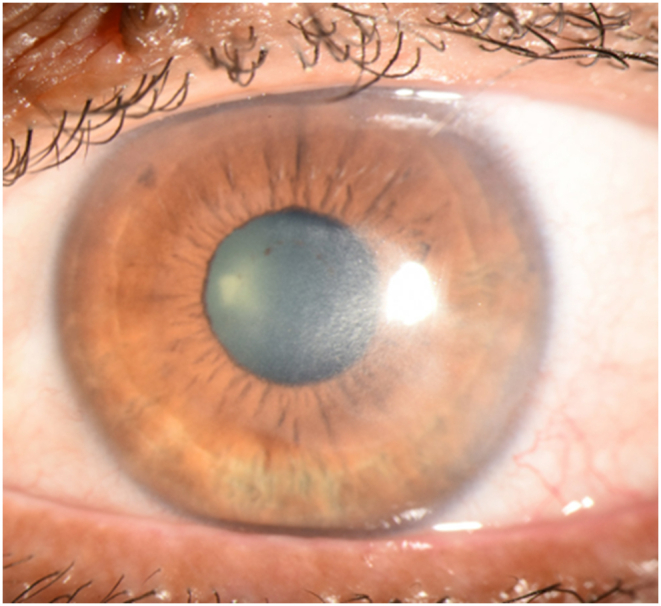
Caption: Slit-lamp photograph of the left eye (December 2019) after treatment with systemic antivirals and topical corticosteroids. The cornea has temporal haze and diffuse, heterogenous stromal scarring without significant involvement of the visual axis.

**Fig. 2:**
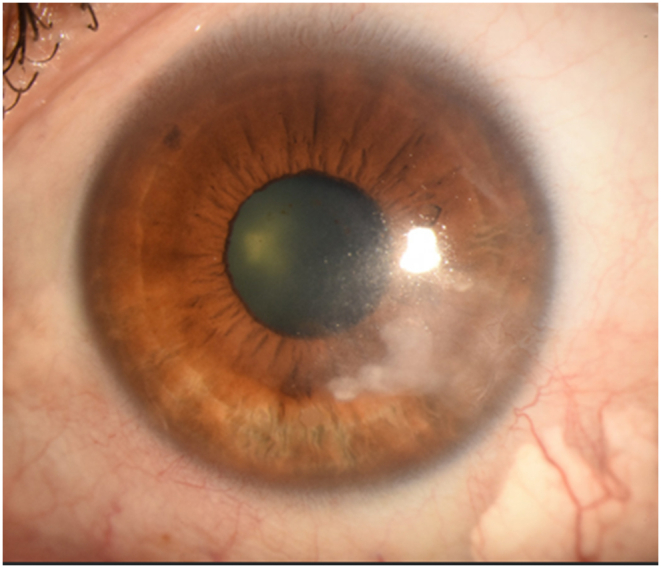
Caption: Slit-lamp photograph (January 2021) taken one month after subconjunctival injection of triamcinolone and bevacizumab. The cornea has interval worsening of lipid keratopathy, haze, and stromal scarring with minimal involvement of the visual axis. Best-corrected visual acuity was 20/30.

A 53-year-old Hispanic male was referred for recurrent herpes simplex virus (HSV) type 1 keratitis in the left eye without corneal NV in September 2019. The best-corrected visual acuity (BCVA) was 20/300. Anterior chamber fluid polymerase chain reaction confirmed HSV-1. The patient was treated with oral acyclovir and loteprednol etabonate 1 % suspension (Lotemax; Bausch and Lomb, Tampa, FL, USA). Three months later, BCVA had improved to 20/80 with residual corneal scarring and haze (Fig. 1). The patient was maintained on acyclovir 400 mg twice daily and loteprednol once daily. One year later (November 2020), the patient had reactivation of keratitis with multiple areas of stromal inflammation with development of minimal corneal NV (pictures not taken at this time). Treatment with oral acyclovir (later switched to oral valacyclovir 1000 mg once daily due to patient preference for once daily dosing and insurance coverage) and topical corticosteroids failed to significantly improve corneal pathology; subconjunctival injections of triamcinolone acetonide (40 mg/mL) and bevacizumab (1.25 mg in 0.05 mL) were performed in December 2020. Follow-up one month later showed residual lipid keratopathy and corneal scarring. Fortuitously, the visual acuity had improved to 20/30, and the patient elected to continue previous maintenance therapy (Fig. 2).

**Fig. 3:**
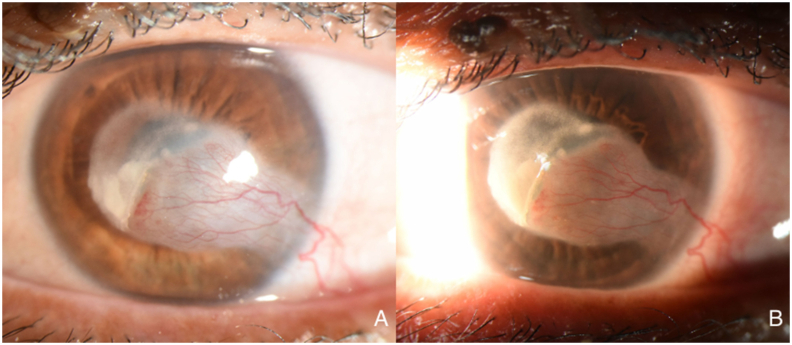
Caption: Slit-lamp photograph taken one year later (January 2022). There is a prominent temporal feeder vessel with regional vascularization and associated lipid deposition with obscuration of the visual axis (Panel A). Sclerotic-scatter view (Panel B) further highlights the heterogeneous nature of lipid deposition and the light-blocking effect of the stromal pathology. Best-corrected visual acuity was 20/400.

The patient was lost to follow-up for one year and returned in January 2022 with a decline of BCVA to 20/400. Clinical examination showed significantly worse corneal NV, “wet” lipid keratopathy (dense, yellow-white infiltrate with light-blocking effect adjacent to corneal NV), and stromal edema encroaching into the visual axis (Fig. 3). The patient was counseled about treatment options, including repeat subconjunctival injections, reinstitution of topical medications, argon laser therapy, FND, and the recent introduction of MICE as a treatment for corneal NV. The patient was amenable to this management approach.

**Fig. 4:**
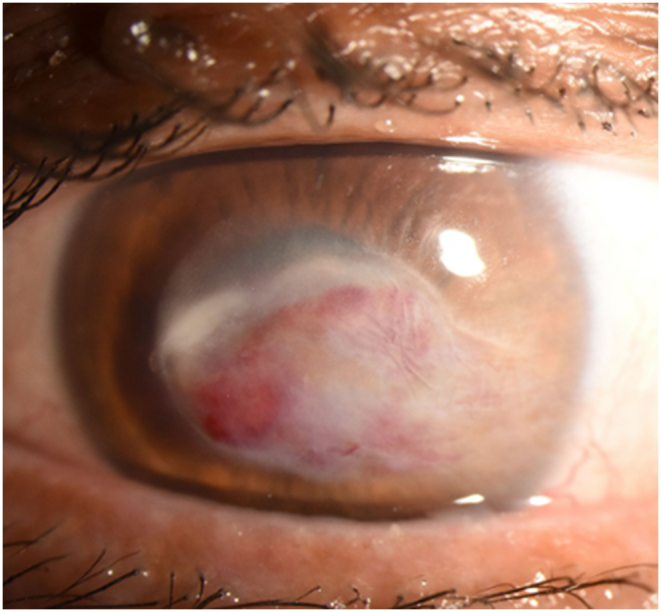
Caption: Slit-lamp photograph taken two weeks after MICE therapy. There is significant regression of the previously seen prominent temporal feeder vessel. There is considerable consolidation of lipids and blood in the corneal stroma resulting in the “pizza pie” appearance of the cornea.

**Fig. 5:**
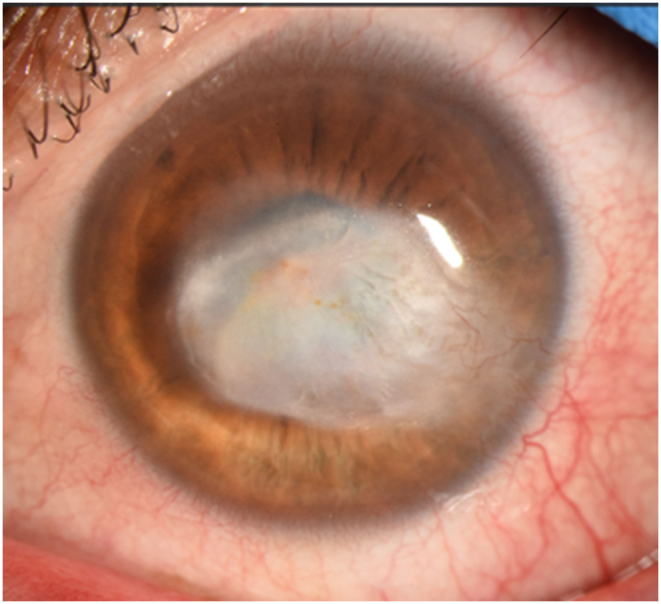
Caption: Slit-lamp photograph taken three months after MICE therapy. The previously seen temporal feeder vessel remains regressed, and there is a significant interval clearing of the lipids and blood seen in Fig. 4. Stromal scarring has consolidated with light-blocking effect encompassing the entire visual axis, obscuring iris and lens details.

MICE was performed in February 2022 using the surgical technique previously described and included as supplementary material (Supplementary Material 1).^7^ The patient was instructed to use topical antibiotics, oral antivirals, and topical corticosteroids postoperatively. Examination two weeks post-MICE demonstrated blood and lipid trapped in the stroma (“pizza-pie cornea”) with noticeable regression of corneal NV (Fig. 4). Oral antivirals and topical corticosteroids were continued twice daily. Three months after MICE (May 2022), there was a prominent reduction in corneal NV and lipid keratopathy; however, stromal scarring that obscured iris details remained, and BCVA continued to be poor (20/200), which was not improved with glasses or contact lenses (Fig. 5).

**Fig. 6:**
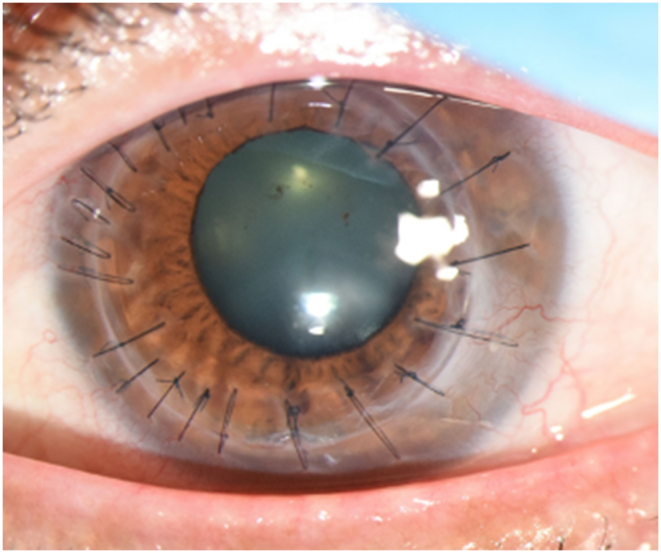
Caption: Slit-lamp photograph taken three months after penetrating keratoplasty demonstrates a clear graft centrally. There is some residual haze and scarring temporal to the graft-host junction in the host cornea with minimal corneal neovascularization. Iris and lens details are appreciated compared to obscuration seen in Fig. 5. The best-corrected visual acuity was 20/40.

**Fig. 7:**
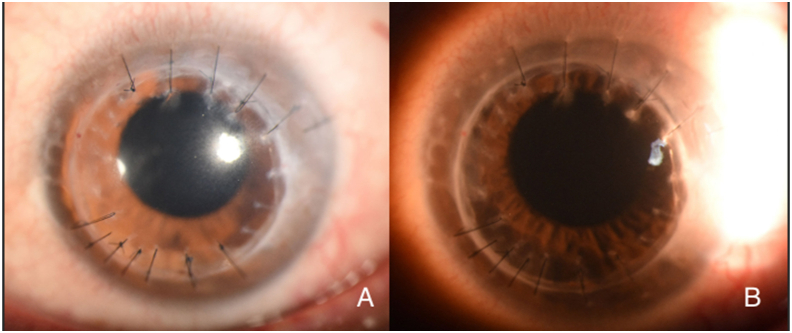
Caption: Slit-lamp photograph taken six weeks after cataract surgery demonstrates a clear penetrating keratoplasty graft centrally. There is continued temporal haze and scarring outside the graft-host junction that has not encroached centrally (Panel A). The sclerotic scatter view (Panel B) further highlights the relative clarity of the central corneal stroma. The best-corrected visual acuity was 20/30.

MICE resulted in the resolution of corneal NV from the central visual axis with some residual corneal NV near the limbus. Due to the remaining scarring limiting BCVA, a decision was made to proceed with corneal transplantation four months after MICE (June 2022). A deep anterior lamellar keratoplasty (DALK) was attempted with the big-bubble technique, but because of large perforation of the Descemet's membrane during bubble creation, a PKP was performed instead of DALK. Three months after PKP, the BCVA improved to 20/40 with a manifest refraction of −1.00 + 1.25 × 135 (Fig. 6). The patient had no further recurrence for six months. Six months after PKP, the patient reported declining visual acuity. Examination at this time revealed BCVA 20/200 and worsening cataract. The patient subsequently underwent cataract surgery with placement of a monofocal IOL in January 2023. The postoperative course was uneventful. Six weeks after cataract surgery, the BCVA was 20/30 with a manifest refraction of −0.75 + 1.25 × 160. Slit-lamp exam revealed a clear PKP without recurrence of corneal NV or lipid deposition (Fig. 7).

Currently, the patient is more than one year post-MICE, one year post-PKP, 6 months post-cataract surgery, and continues to maintain a 20/30 BCVA without recurrence of corneal NV. The patient continues to take oral valacyclovir 1000 mg once daily and topical prednisolone acetate 1 % once daily.

## 3 Discussion

Corneal NV is a sight-threatening condition characterized by the abnormal growth of blood vessels in the cornea and is a leading cause of corneal blindness.^1^^,^^2^ While the incidence of corneal NV is on the rise, effective treatment strategies remain lacking.^3^^,^^8^ MICE is a recently-introduced and favorable option for the treatment of primary corneal NV.^7^ In this case report, we offer that the role of MICE can be expanded into a prophylactic therapy to treat corneal NV for eyes that are otherwise at high risk for surgical failure and rejection with PKP.

Corneal avascularity is an active process involving the production of antiangiogenic factors to counterbalance proangiogenic factors, including VEGF.^9^ Current treatment options for corneal NV, including corticosteroids, anti-VEGF, and argon laser, have been limited in their ability to treat the underlying cause of corneal NV.^3^^,^^8^^,^^10^ While VEGF is prominent in angiogenesis and neovascular ocular diseases, anti-VEGF treatment options are effective against corneal NV only when administered early in the disease process and only target VEGF-A.^11^ Moreover, the presence of pericytes around corneal NV vessels as a barrier further diminishes the effectiveness of anti-VEGF options.^12^ Challenges with laser-induced photocoagulation include difficulty in identifying feeder vessels, upregulation of VEGF, and long-term regression without maintenance treatment.^3^^,^^8^ Although combination therapy with the above options has shown some success in reducing corneal NV, often the improvement is short-term with relatively quick recurrence of the neovascularization.^10^ Fine-needle diathermy, with or without subconjunctival bevacizumab, as a primary therapy or prophylactic to keratoplasty may also be employed as a strategy in these cases, with several groups reporting favorable results.^13^, ^14^, ^15^

Unlike other treatments, MICE offers a potential solution by harnessing the irreversibly cytotoxic effects of MMC on endothelial cells of the systemic vasculature.^16^^,^^17^ An ideal candidate eye for MICE is one with lipid keratopathy secondary to corneal NV threatening or affecting the visual axis, controlled underlying etiology, an inflamed eye, and an unsuccessful trial of topical corticosteroids.^7^ However, it is important to note that MICE is a technically demanding procedure, requiring meticulous attention to the injection site and proper angling of the needle (Ouano D, written communication, February 4, 2022). Additionally, the potential corneal toxicity caused by MMC, particularly to the corneal endothelium, should also be given consideration.^18^ Nevertheless, encouraging results of MICE have been reported, suggesting a favorable lack of short-term adverse effects and the preservation of non-affected limbic vascularization.^7^ However, we still advise surgeons to be mindful of potential toxicity to the corneal endothelium from MMC, as previously and extensively described in the literature. Serial examinations of post-MICE patients, with objective assessments of corneal endothelium with confocal or specular microscopy, should be considered.

In our patient, we used MICE as a prophylactic therapy to treat corneal NV in an eye that would have otherwise had poor success with PKP. Surgical success with corneal transplantation in the presence of florid, active corneal NV is considerably less favorable than in eyes with non-vascularized pathologies, with rates of rejection ranging from 3 to 36 % across a period of 1–10 years postoperatively.^6^ Experienced corneal surgeons are also likely familiar with intraoperative challenges in highly vascularized eyes, such as surgical field bleeding and decreased visualization of the ocular surface, which increases the difficulty of corneal transplant surgery. Additionally, we noted difficulty in creating an appropriate big bubble for DALK in our patient, which led to conversion to PKP. It is possible that the scarring, either from the initial corneal NV process or MICE-induced stromal changes, led to these difficulties. Additional experiences are warranted to explore our experience.

We were able to perform MICE to eliminate the prominent feeder vessels and decrease the stromal lipid deposition. This created a favorable environment such that even though the poor BCVA and corneal scarring remained, we were later able to perform PKP successfully. More than one year after MICE, the patient did not have a recurrence of corneal NV despite two subsequent ocular surgeries (PKP and cataract surgery) and has maintained a favorable BCVA. We note that the patient remained on antivirals and topical steroids, which may have additionally contributed to the lack of corneal NV recurrence. We also note that the peripheral vessels in our patient may represent a factor for graft rejection and other complications in the future; thus, patients after MICE need to be monitored closely, including consideration for additional or alternative treatments, to ensure long-term graft success. Although further research is necessary to optimize suitable candidates and evaluate the long-term effects of MICE, our case contributes to the growing body of evidence supporting the efficacy of MICE not only as a primary treatment but also as a valuable prophylactic intervention for corneal NV in eyes that may require additional surgical interventions. The long-term success rate of PKP after MICE, especially given significantly higher rejection rates in vascularized corneas,^6^ remains to be seen.

After conducting a literature review on June 10, 2023 utilizing PubMed and Google Scholar, using the keywords “MICE”, “mitomycin intravascular chemoembolization”, “penetrating keratoplasty”, and “corneal neovascularization”, we did not find any prior reports of the use of MICE as a prophylactic treatment for corneal NV before subsequent corneal transplantation. We propose that MICE can successfully treat corneal NV secondary to HSV-1 in high-risk eyes and create a favorable environment for successful PKP with good short-term results.

## Patient consent

The patient consented to the publication of this case report in writing.

## Funding

This study did not have any sources of funding.

## Authorship

All authors attest that they meet the current ICMJE criteria for authorship.

## CRediT authorship contribution statement

**Neal Rangu:** Writing – original draft, Methodology, Investigation, Data curation. **Kamran M. Riaz:** Writing – review & editing, Writing – original draft, Supervision, Resources, Project administration, Formal analysis, Data curation, Conceptualization.

## Declaration of competing interest

The authors declare that they have no known competing financial interests or personal relationships that could have appeared to influence the work reported in this paper.

## References

[1] Lasagni Vitar R.M., Triolo G., Fonteyne P. (2021). Epidemiology of corneal neovascularization and its impact on visual acuity and sensitivity: a 14-year retrospective study. Front Med.

[2] Abdelfattah N.S., Amgad M., Zayed A.A. (2015). Clinical correlates of common corneal neovascular diseases: a literature review. Int J Ophthalmol.

[3] Feizi S., Azari A.A., Safapour S. (2017). Therapeutic approaches for corneal neovascularization. Eye Vis (Lond).

[4] Cursiefen C., Wenkel H., Martus P. (2001). Impact of short-term versus long-term topical steroids on corneal neovascularization after non-high-risk keratoplasty. Graefes Arch Clin Exp Ophthalmol.

[5] Chu H.S., Chen T.C., Hu F.R., Chen W.L. (2013). Recurrence of corneal neovascularization associated with lipid deposition after subconjunctival injection of bevacizumab. Cornea.

[6] Bachmann B., Taylor R.S., Cursiefen C. (2010). Corneal neovascularization as a risk factor for graft failure and rejection after keratoplasty: an evidence-based meta-analysis. Ophthalmology.

[7] Mimouni M., Ouano D. (2022). Initial outcomes of mitomycin intravascular chemoembolization (MICE) for corneal neovascularization. Int Ophthalmol.

[8] Gupta D., Illingworth C. (2011). Treatments for corneal neovascularization: a review. Cornea.

[9] Azar D.T. (2006). Corneal angiogenic privilege: angiogenic and antiangiogenic factors in corneal avascularity, vasculogenesis, and wound healing (an American Ophthalmological Society thesis). Trans Am Ophthalmol Soc.

[10] Anand N., Reidy J.J., Riaz K.M. (2019). Short-term regression of corneal neovascularization with combination therapy of argon green laser photocoagulation and subconjunctival bevacizumab. Int Med Case Rep J.

[11] Stevenson W., Cheng S.F., Dastjerdi M.H., Ferrari G., Dana R. (2012). Corneal neovascularization and the utility of topical VEGF inhibition: ranibizumab (Lucentis) vs bevacizumab (Avastin). Ocul Surf.

[12] Cursiefen C., Hofmann-Rummelt C., Küchle M., Schlötzer-Schrehardt U. (2003). Pericyte recruitment in human corneal angiogenesis: an ultrastructural study with clinicopathological correlation. Br J Ophthalmol.

[13] Faraj L.A., Elalfy M.S., Said D.G., Dua H.S. (2014). Fine needle diathermy occlusion of corneal vessels. Br J Ophthalmol.

[14] Koenig Y., Bock F., Kruse F.E., Stock K., Cursiefen C. (2012). Angioregressive pretreatment of mature corneal blood vessels before keratoplasty: fine-needle vessel coagulation combined with anti-VEGFs. Cornea.

[15] Mestanoglu M., Händel A., Cursiefen C., Hos D. (2022). Three-year follow-up of high-risk keratoplasty following fine-needle diathermy of corneal neovascularization combined with bevacizumab. Graefes Arch Clin Exp Ophthalmol.

[16] Hoorn C.M., Wagner J.G., Petry T.W., Roth R.A. (1995). Toxicity of mitomycin C toward cultured pulmonary artery endothelium. Toxicol Appl Pharmacol.

[17] Seki Y., Toba K., Fuse I. (2005). In vitro effect of cyclosporin A, mitomycin C and prednisolone on cell kinetics in cultured human umbilical vein endothelial cells. Thromb Res.

[18] Goldsberry D.H., Epstein R.J., Majmudar P.A. (2007). Effect of mitomycin C on the corneal endothelium when used for corneal subepithelial haze prophylaxis following photorefractive keratectomy. J Refract Surg.

