# Supplementary material for: Mitomycin intravascular chemoembolization (MICE) to treat corneal vascularization prior to penetrating keratoplasty
Source: Am J Ophthalmol Case Rep. 2024 Jan 14;33:101993. doi: 10.1016/j.ajoc.2024.101993 (PMC10828578; doi:10.1016/j.ajoc.2024.101993)
Supplement: Multimedia component 1 [file mmc1.docx]

**Mitomycin Intravascular Chemo-Embolization (MICE)**

Technique first described by Dean Ouano, MD and Michael Mimouni, MD: Mimouni M, Ouano D. Initial outcomes of mitomycin intravascular chemoembolization (MICE) for corneal neovascularization. Int Ophthalmol. 2022 Aug;42(8):2407-2416.

*The following is an adaptation and summary of the MICE procedure*

Ideal Candidate for MICE

A. Patient with lipid keratopathy secondary to corneal NV encroaching on the visual axis (once the visual axis is significantly involved, the prognosis is less favorable)
B. Underlying etiology controlled (HSV/lid margin disease etc..)
C. Quiet and uninflamed eye
D. Failed trial of topical steroids (and antivirals if herpes related)

Preoperative Planning (suggested clinical documentation and assessment)

- Slit-lamp photos
- Corneal topography
- Corneal tomography
- Anterior segment OCT
- Assessment of corneal sensation
- Corneal pachymetry (ultrasonic)

Injection Protocol

- TSK thin-walled 33-G needle or JPB 34-G Nanoneedle with partially filled 1.0 cc "luer slip” not luer lock syringe.
  - Avoid using 30g or 32g needle as the needle may be too big to cannulate small corneal vessels
- Mitomycin at a concentration of 0.3-0.4 mg/ml 🡪 injecting 0.01 to 0.05 mL means that maximum of 0.02 mg is injected (safety concerns)
- Aim for the largest bore corneal vessel(s) inside the limbus
- Inject with enough FORCE to get into both efferent/afferent vessels 🡪 Low angle of penetration (~15 deg) to cannulate the vessel
- Look for a “flash” once inside the vessel
- Can give multiple treatments to vessels – look for blanching

Links to Videos

MICE: Intrastromal Misses and Intravascular Hits - <https://www.youtube.com/watch?v=45UBsOK6w2Q>

MICE: How to Video - <https://www.youtube.com/watch?v=zcJCv_sCerA&t=1s>

Pulse and hold MICE - <https://www.youtube.com/watch?v=IIVMe9E2w3E>

Riaz MICE Video 1 - <https://youtu.be/UUgP8z42sTk>

Postoperative Management

Topical antibiotics and steroids QID. Antibiotics to be stopped 7-10 days after procedure. Steroids to be tapered over 1 month or per surgeon’s discretion

*Expected findings*

POD1-7: blanched/ablated vessels

POW1-2: “pizza pie sign”: blood and lipid trapped in corneal stroma


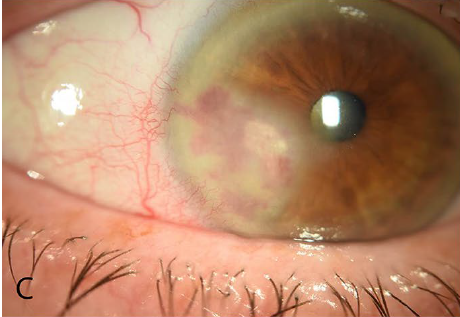


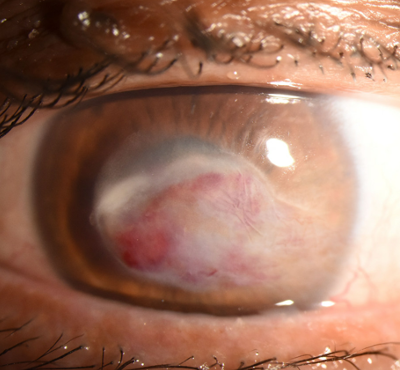


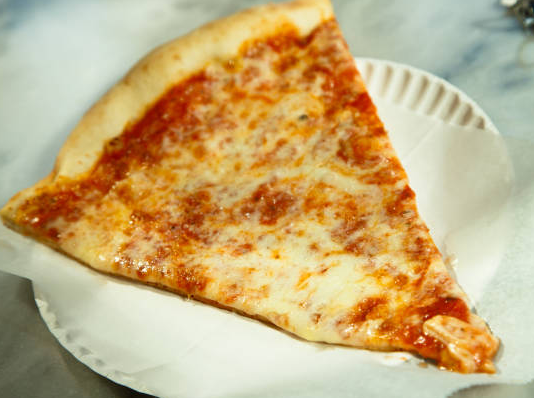


POM1-3: lipid absorption leading to a “feathery” appearance of lipid with concurrent stromal compaction. AS-OCT may be helpful to visualize stromal compaction. Astigmatism may occur at this time. Comparison with preoperative tomography may be helpful.
